# Prophylactic doxazosin reduces urinary retention and promotes recovery after total joint arthroplasty: A randomized controlled trial

**DOI:** 10.3389/fphar.2022.1016203

**Published:** 2023-01-09

**Authors:** Zichuan Ding, Jian Cao, Chao Huang, Kai Zhou, Haoyang Wang, Zongke Zhou

**Affiliations:** Department of Orthopedics, West China Hospital, Sichuan University, Chengdu, Sichuan, China

**Keywords:** doxazosin, postoperative urinary retention, total joint arthroplasty, postoperative recovery, randomized controlled trial

## Abstract

**Background:** Postoperative urinary retention (POUR) is a common and disruptive complication following total joint arthroplasty (TJA). The aim of this study is to investigate whether doxazosin can decrease the incidence of POUR and promote recovery under the setting of modern enhanced recovery after TJA.

**Methods:** In this randomized placebo-controlled trial, patients over 35 years of age undergoing primary unilateral TJA were recruited. Patients received doxazosin (4 mg once) or placebo 2 h before surgery. The primary outcome of interest was the development of POUR, which was diagnosed when patients with a urine volume over 400 ml or overflow incontinence. Postoperative recovery was assessed in terms of hospital length of stay after surgery, daily ambulation distance, visual analogue scale (VAS) pain score and opioid consumption.

**Results:** A total of 170 male patients were equally randomized into Doxazosin group (mean age 54.2 ± 13.7 years, range 36–88 years) and Placebo group (mean age 54.6 ± 13.9 years, range 38–81 years). The POUR rate was significant lower in Doxazosin group (17.6%) than in Placebo group (36.5%) (*p* = .006). The mean LOS in the Doxazosin group was 3.1 ± 1.1 days compared to 3.6 ± 1.7 days in the Placebo group (*p* = .030). Doxazosin group had a longer daily mobilization distance than Placebo group on postoperative day 1 (26.8 ± 11.1 vs. 22.8 ± 9.7; *p* = .015). Postoperative pain assessed by VAS score and opioid usage was comparable between two groups.

**Conclusion:** Our results support the routine use of prophylactic doxazosin in male patients to decrease POUR rate and promote postoperative recovery under the setting of modern enhanced recovery after TJA.

## Introduction

Postoperative urinary retention (POUR) is common following the total joint arthroplasty (TJA), with reported rates ranging from 20.0% to 46.3% in the literatures (Bjerregaard et al., 2015; Scholten et al., 2018; Schubert et al., 2019; Bracey et al., 2021). As a disruptive complication, POUR can lead to overdistension of the bladder in 44% of cases, which may ultimately result in urologic injury and infection (Baldini et al., 2009). Besides, POUR may lead to the increase in urea and creatinine, which can even result in serious cardiovascular problems, especially in the elderly population. Since indwelling or intermittent urinary catheterization is the only effective management for POUR, the risk of catheter-related complications, including urinary tract infection, bacteremia and even periprosthetic joint infection, can be further increased (Pulido et al., 2008; Nicolle, 2014). Besides, POUR and urinary catheterization are risk factors of increased postoperative pain, reduced early mobilization and prolonged hospital length of stay (LOS) (Pivec et al., 2021; Bracey et al., 2022). POUR severely affects the postoperative recovery after TJA, which is unacceptable for both patients and surgeons under the current setting of modern enhanced recovery after TJA.

Although POUR is far from a new phenomenon and its importance has been entirely highlighted, very few approaches have developed to mitigate its occurrence (Cha et al., 2020; Bracey et al., 2022). Pharmacologic intervention has been considered to be the most effective and direct way to prevent POUR and reduce the use of catheterization after surgery. Alpha adrenoreceptor blockers, such as tamsulosin, doxazosin and prazosin, are the first-line drugs for the lower urinary tract symptoms treatment. These medications can decrease the resistance of the urethra by decreasing the tension of smooth muscle in the prostate and bladder neck, which are rich in alpha1-adrenergic receptors. As a result, alpha adrenoreceptor blockers are proposed to reduce the incidence of POUR after TJA (Balderi and Carli, 2010).

A retrospective study recruiting 559 TJA male patients found that patients who received alpha adrenoreceptor blockers perioperatively had significantly lower risk of POUR and reduced LOS (Schubert et al., 2020). Though some randomized controlled trials have compared the efficacy of tamsulosin and placebo to prevent POUR after TJA, the results seemed controversial (Schubert et al., 2019; Choi et al., 2021). Schubert et al. found no difference in POUR incidence between prophylactic tamsulosin and placebo after TJA (4), while Choi et al. (2021) drew an opposite conclusion. Although Choi et al. (2021)’s study is more recent than Schubert et al. (2019)‘s study, both investigations were of high quality and cannot be ignored. Besides, the perioperative managements in these trials, such as placing indwelling catheters intraoperatively, did not conform to the concept of modern enhanced recovery after TJA and may contribute to the development of POUR and confound the results (Choi et al., 2021).

Doxazosin gastrointestinal therapeutic system and tamsulosin are the most frequently prescribed two alpha adrenoreceptor blockers, and doxazosin gastrointestinal therapeutic system has been proven to be more efficient than tamsulosin in treating lower urinary tract symptoms (Guo and Tang, 2021). As a result, we conducted this randomized placebo-controlled trial to investigate whether doxazosin can decrease the rate of POUR and promote recovery under the setting of modern enhanced recovery after TJA.

## Materials and methods

The Ethical Committee of our institution approved this randomized controlled clinical trial. The registration number of our study at the Chinese Clinical Trial Registry was ChiCTR2200056992. Written informed consents were obtained from all participants prior to the intervention.

### Participants recruitment and randomization

Male patients over 35 years of age who were scheduled for primary unilateral total hip/knee arthroplasty from February 2022 to June 2022 were included in this study. Exclusion criteria included American Society of Anesthesiologists (ASA) status IV, use of preoperative or intraoperative urinary catheter, allergy or intolerance to doxazosin, exposure to alpha-blocking medication or 5-alpha reductase inhibitor medication or anticholinergic medications within 30 days prior to the surgery, impaired liver/renal function, opioid dependence, a history of hypotension, a history of myocardial infarction and a history of gastrointestinal strictures.

An independent research assistant performed the randomization using a computer-generated randomization table. The randomization allocation was concealed in sealed opaque envelopes with consecutive numbers. Indistinguishable tablets containing either doxazosin or placebo were prepared by a dedicated study nurse. After envelopes were opened, patients received a single dose of doxazosin (4 mg once, doxazosin gastrointestinal therapeutic system) or placebo 2 h before surgery. Patients, surgeons and investigators were all blinded to the allocation until the data analysis was completed.

### Perioperative management

All surgeries were performed by two senior surgeons under general anesthesia without the preoperative or intraoperative use of urinary catheter. Sufentanil .5 μg/kg, midazolam .04 mg/kg, propofol 1–2 mg/kg and cistracurium 2 μg/kg intravenously were used for anesthesia induction, and a following continuous intravenous infusion of .1–.3 μg/(kgmin) of remifentanil, 2–5 mg/(kgh) of propofol and inhalation of sevoflurane were used for anesthesia maintenance (Ding et al., 2020). After surgery, the development of POUR was monitored by the clinical symptoms and bladder ultrasound scans. Patients who failed to void within 4 h after surgery or had clinical symptoms, including suprapubic discomfort, distention symptoms or urinary incontinence, were assessed with the urine volumes. Patients with urine volumes over 400 ml were encouraged to get off the bed and void, and patients still unable to void were managed with a straight catheterization. Patients with urine volumes less than 400 ml had repeated assessment in 2 hours. All participants accepted the same perioperative multimodal analgesic protocol as previous reported (Ding et al., 2022a).

### Outcomes

The primary outcome of interest was the development of POUR, which was diagnosed when patients with a urine volume over 400 ml or overflow incontinence. Postoperative recovery was assessed in terms of hospital LOS after surgery, daily ambulation distance, visual analogue scale (VAS) pain score, hospitalization opioid consumption, blood pressure and C-reactive protein. Urinary tract infection and other complications including hypotension, dizziness, nausea and vomiting, fatigue, pruritis, wound infection, delayed wound healing and venous thrombosis, were also recorded (Luo et al., 2018). Patients were followed up until discharge.

### Sample size calculation and statistical analysis

The sample size was calculated depending on the primary outcome of POUR incidence. According to previous publications, the POUR rate of male patients after TJA was around 40%, and a reduction of 50% (POUR rate of 20% in experiment group) can be regarded as a clinically meaningful difference (Bjerregaard et al., 2015; Scholten et al., 2018). Therefore, at least a sample size of 79 patients in each group would be needed, with a 2-sided alpha level of 5% and a power of 80%.

The normality of data was measured with histograms and quantile-quantile plots. Continuous data with normal distribution were analyzed using the Student’s t-test. Continuous data with skewed distribution were compared using the Mann-Whitney *U* test. Categorical data are expressed as the number and percentage and were analyzed using Pearson chi-square test or Fisher exact test as appropriate. The level of significance was set at *p*-value <.05. Statistical analysis was performed using SPSS v22.0 (IBM, Armonk, NY).

## Results

**FIGURE 1:**
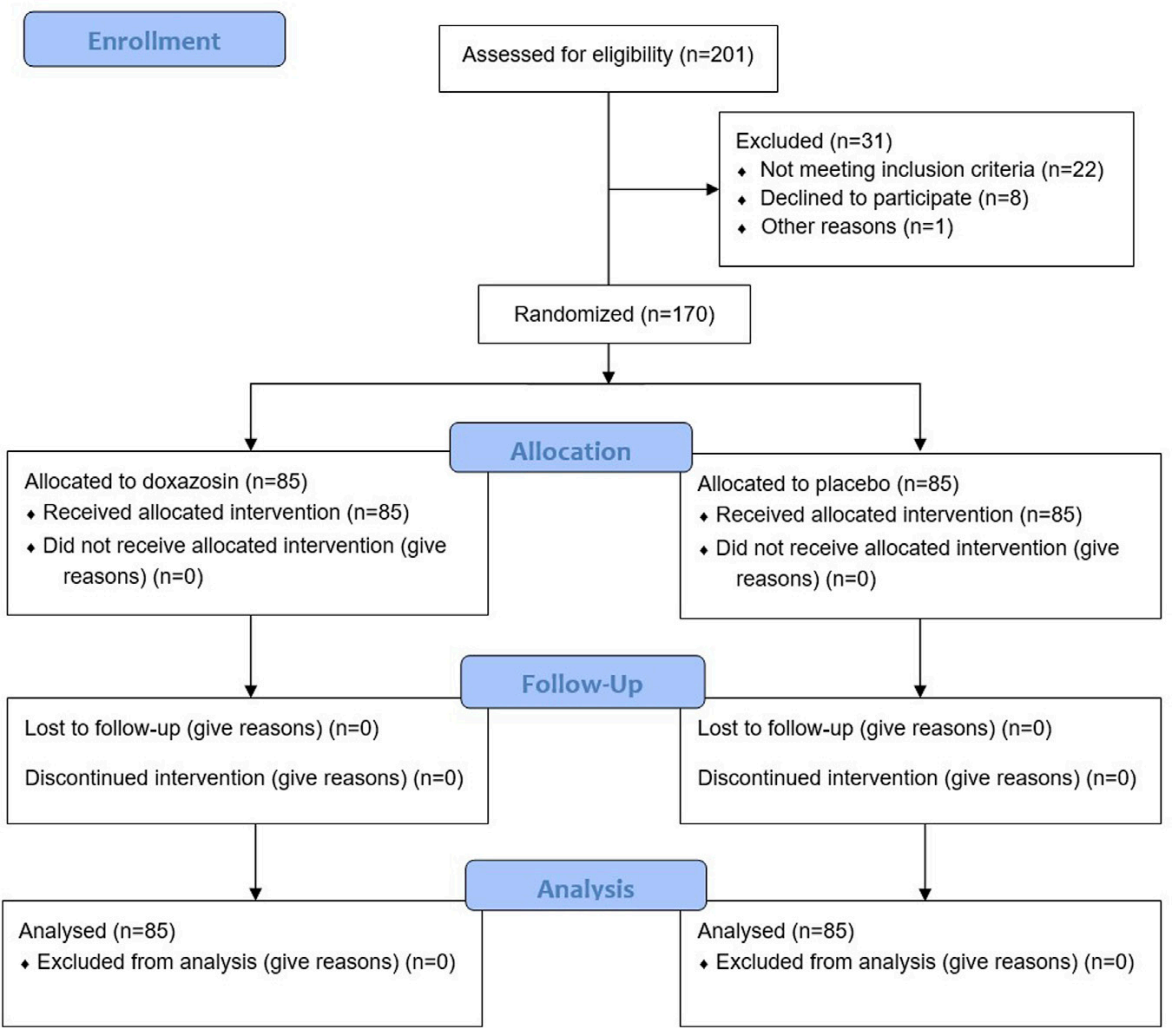
CONSORT flow diagram.

A total of 201 male patients were assessed for eligibility, of whom 31 patients were excluded for reasons (Figure 1). The remaining 170 patients were equally randomized into Doxazosin group and Placebo group. No patients dropped out of the study during the follow-up. The demographics of the enrolled patients showed no significant difference between two groups (Table 1).

**TABLE 1:** Demographics.

Variable	Doxazosin (*n* = 85)	Placebo (*n* = 85)	*p*
Mean age (range)	54.2 ± 13.7 (36–88)	54.6 ± 13.9 (38–81)	.842
Sex, male	85 (100.0%)	85 (100.0%)	1.000
Height (cm)	167.8 ± 6.5	165.9 ± 7.6	.085
Weight (kg)	68.1 ± 10.7	69.0 ± 19.1	.710
BMI	24.1 ± 3.3	24.9 ± 6.8	.326
Surgery type			1.000
THA	68 (80.0%)	68 (80.0%)	
TKA	17 (20.0%)	17 (20.0%)	
Diagnosis			.956
ONFH	49 (57.6%)	46 (54.1%)	
DDH	5 (5.9%)	6 (7.1%)	
Hip osteoarthritis	14 (16.5%)	16 (18.8%)	
Knee osteoarthritis	17 (20.0%)	17 (20.0%)	
Side, left	41 (48.2%)	42 (49.4%)	.878
Intraoperative intravenous fluid (ml)	895.0 ± 307.6	968.3 ± 321.6	.146
Duration of the surgery (min)	128.2 ± 29.9	133.8 ± 35.4	.288
Blood pressure			
Systolic pressure (mmHg)	124.8 ± 17.7	121.6 ± 18.1	.246
Diastolic pressure (mmHg)	77.4 ± 10.5	75.3 ± 9.9	.181
IPSS score	8.1 ± 7.0	7.4 ± 4.9	.488
IPSS grade			.508
I	46 (54.1%)	47 (55.3%)	
II	34 (40.0%)	36 (42.4%)	
III	5 (5.9%)	2 (2.3%)	
History of urinary retention	9 (10.6%)	8 (9.4%)	.798
History of urinary system diseases	16 (18.8%)	12 (14.1%)	.408

Continuous variables are presented as mean ± SD., Categorical variables are presented as number with frequency (%). BMI, body mass index; THA, total hip arthroplasty; TKA, total knee arthroplasty; ONFH, osteonecrosis of the femoral head; DDH, developmental dysplasia of hip; IPSS, international prostate symptom score.

**FIGURE 2:**
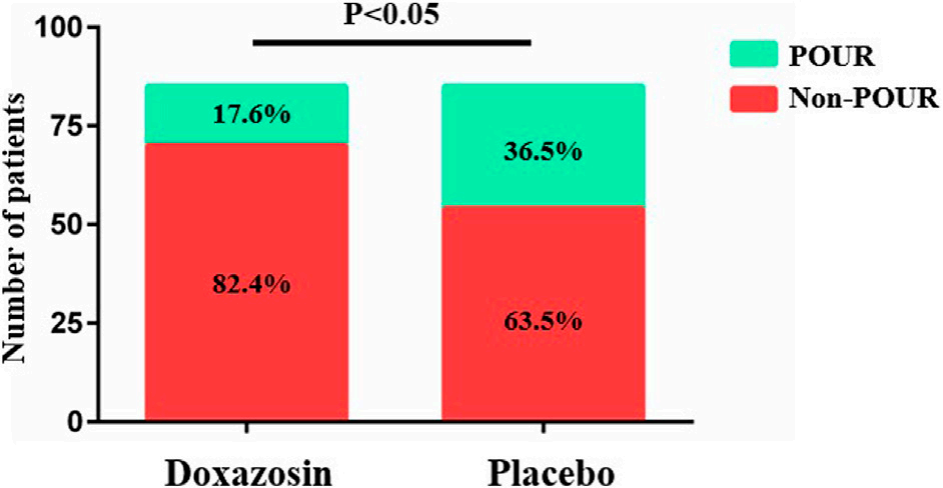
Number of patients developing POUR.

The results regarding POUR and postoperative recovery are presented in Table 2. A total of 46 patients (27.1%) developed POUR in the entire cohort of patients, and the POUR rate was significantly lower in Doxazosin group (15 out of 85; 17.6%) than in Placebo group (31 out of 85; 36.5%) (*p* = .006) (Figure 2). Three patients received repeated catheterization in Doxazosin group and seven patients needed repeated catheterization in Placebo group. All the catheters were discontinued prior to discharge. The mean LOS in the Doxazosin group was 3.1 days compared to 3.6 days in the Placebo group (*p* = .030). Doxazosin group had a longer daily mobilization distance than Placebo group on postoperative day 1 (26.8 ± 11.1 vs. 22.8 ± 9.7; *p* = .015). Postoperative pain assessed by VAS score and opioid usage was comparable between two groups. The rates of urinary tract infection were very low both in Doxazosin group and Placebo group, with no statistical significance (1.2% vs. 2.4%; *p* = 1.000).

**TABLE 2:** Outcomes.

Variable	Doxazosin (*n* = 85)	Placebo (*n* = 85)	*p*
Postoperative urinary retention	15 (17.6%)	31 (36.5%)	.006
Length of stay	3.1 ± 1.1	3.6 ± 1.7	.030
Daily mobilization m)			
Day 1	26.8 ± 11.1	22.8 ± 9.7	.015
Day 2	38.8 ± 12.8	35.2 ± 14.2	.089
VAS			
Day 1	3.8 ± 1.3	4.0 ± 1.5	.335
Day 2	2.8 ± 1.1	2.9 ± 1.4	.515
Opioid usage (mg)	12.8 ± 8.7	13.0 ± 8.6	.930
Urinary tract infection	1 (1.2%)	2 (2.4%)	1.000
Blood pressure			
Systolic pressure (mmHg)	141.1 ± 20.6	80.4 ± 11.3	.661
Diastolic pressure (mmHg)	142.5 ± 21.0	81.2 ± 11.8	.652
C-reactive protein	27.5 ± 15.1	29.7 ± 15.4	.348

Continuous variables are presented as mean ± SD., Categorical variables are presented as number with frequency (%).

Table 3 shows the incidence of possible adverse events related to doxazosin and postoperative complications. No significant difference was observed in adverse events between groups, such as hypotension, dizziness, nausea and vomiting, fatigue and pruritis. The rates of other postoperative complications were also similar between groups. All adverse events and complications were successfully treated without cessation or patient death.

**TABLE 3:** Adverse events.

	Doxazosin (*n* = 85)	Placebo (*n* = 85)	*p*
Hypotension	3 (3.5%)	1 (1.2%)	.621
Dizziness	2 (2.4%)	0 (0%)	.497
Nausea and vomiting	2 (2.4%)	3 (3.5%)	1.000
Fatigue	2 (2.4%)	2 (2.4%)	1.000
Pruritis	1 (1.2%)	3 (3.5%)	.621
Wound infection	0 (0%)	0 (0%)	—
Delayed wound healing	7 (8.2%)	5 (5.9%)	.549
Deep vein thrombosis	0 (0%)	0 (0%)	—
Periprosthetic joint infection	0 (0%)	0 (0%)	—
Blood transfusion	1 (1.2%)	1 (1.2%)	1.000
Neurovascular events	1 (1.2%)	0 (0%)	1.000

Data are presented as number with frequency (%) and compared with Pearson chi-square test or Fisher exact test.

## Discussion

POUR, a common and disruptive complication following TJA, can severely affect the postoperative recovery after TJA and increase the risk of other postoperative complications (Bracey et al., 2022). There remains no efficient approach to mitigating POUR and avoiding the use of indwelling or intermittent urinary catheterization for the treatment of POUR. The most valuable finding of this randomized, double-blind, placebo-controlled trial was that doxazosin 4 mg once (2 h before surgery) can decrease the rate of POUR, increase ambulation distance and reduce hospital LOS. Our results support the routine use of prophylactic single-dose doxazosin in male patients to decrease the rate of POUR and promote postoperative recovery under the setting of modern enhanced recovery after TJA.

In this study, the overall POUR rate in the entire cohort of patients was 27.1% (17.6% in Doxazosin group and 36.5% in Placebo group), which accords with the previous publications and confirms the importance of prevention for this high-incidence complication (Bjerregaard et al., 2015; Scholten et al., 2018; Pivec et al., 2021). It is worth mentioning that the rate of POUR after TJA can be highly variable in the literatures, from 5.1% to 46.3% (Bjerregaard et al., 2015; Scholten et al., 2018; Pivec et al., 2021). The reason for the varied incidence may be that the definitions of POUR were quite different among studies. Some studies used bladder scan to assess the volume of urine in the bladder, and POUR was diagnosed if urine volume exceeds 400 ml (Scotting et al., 2019; Bracey et al., 2021). Some studies relied on the subjective patient reports and the clinical symptoms, such as suprapubic discomfort, distention symptoms and urinary incontinence, to define POUR (Santini et al., 2019; Abdul-Muhsin et al., 2020). It is obvious that bladder scan has higher accuracy and sensitivity than subjective patient reports, and investigations performing bladder scan would expectedly have higher incidences of developing POUR.

Since the only treatment option for POUR is urethral catheterization, and either intermittent or indwelling catheterization can impede the postoperative recovery and increase risk of complications, researchers have been focusing on the prevention of POUR. Alpha adrenoreceptor blockers have been considered to be able to prevent the occurrence of POUR based on the mechanism of decreasing the tension of smooth muscle in the urethra and their use in the treatment of benign prostate hyperplasia. A well-designed double-blinded randomized controlled trial used tamsulosin to prevent POUR in male patients after TJA. They found an approximately 30% decrease of POUR rate in the tamsulosin group, but it did not reach statistically significant (Schubert et al., 2019). On the contrary, our study found doxazosin could lower the risk of POUR in male patients over 35 years old, who are at higher risk of developing POUR (Fernandez et al., 2014; Ziemba-Davis et al., 2019). The superior pharmacokinetic profile and drug delivery rate of doxazosin gastrointestinal therapeutic system used in this study may explain the differences in the findings (Guo and Tang, 2021). Besides, our results were in accordance with a recent randomized controlled study (Choi et al., 2021) and two earlier prospective studies (Tammela et al., 1987; Petersen et al., 1991) confirming the preventive effect of alpha adrenoreceptor blockers for POUR.

Enhancing the postoperative recovery at early stage is the critical point of modern TJA (Ding et al., 2022b). Early mobilization ability and LOS are the most frequently used outcomes to measure the postoperative recovery effect after TJA (Soffin and YaDeau, 2016). Our results showed that doxazosin could increase the mobilization on the first postoperative day and decrease LOS when compared to the placebo. POUR has been demonstrated to impact postoperative recovery (Pivec et al., 2021; Bracey et al., 2022), and remarkably, doxazosin could promote the recovery through decreasing the risk of POUR. The results were similar to a propensity-matched retrospective cohort study which found the cohort taking alpha adrenoreceptor blockers had a 12.1% decreased risk of POUR and a one-day reduced LOS. Besides, no significant side effect of doxazosin, such as hypotension, dizziness, nausea and vomiting, fatigue and pruritis, was observed in this study, possibly due to the low clinical dosage and short-term treatment of doxazosin.

Several limitations existed in our study. Firstly, the perioperative management, especially the urinary tract management, can be quite variable across different institutions, which may affect the generalizability of our results. Secondly, all patients in this study received general anesthesia, which was reported to be at lower risk of POUR than spinal anesthesia in general surgery (Brouwer et al., 2021). General anesthesia may influence the incidence of POUR and thus influence the preventive effect of doxazosin on POUR in this study. Thirdly, patients were asked to void prior to surgery and we did not assess the urine volume prior to surgery. Measurement of postvoid residual prior to surgery may better estimate the incidence of preoperative urinary retention.

## Conclusion

In conclusion, prophylactic single-dose doxazosin reduced the incidence of POUR, increased ambulation distance and reduced hospital LOS after TJA. Our results support the routine use of prophylactic doxazosin in male patients to decrease the rate of POUR and promote postoperative recovery under the setting of modern enhanced recovery after TJA.

## Data Availability

The raw data supporting the conclusion of this article will be made available by the authors, without undue reservation.
